# Large-Scale Mitochondrial DNA Analysis of the Domestic Goat Reveals Six Haplogroups with High Diversity

**DOI:** 10.1371/journal.pone.0001012

**Published:** 2007-10-10

**Authors:** Saeid Naderi, Hamid-Reza Rezaei, Pierre Taberlet, Stéphanie Zundel, Seyed-Abbas Rafat, Hamid-Reza Naghash, Mohamed A. A. El-Barody, Okan Ertugrul, François Pompanon, for the Econogene Consortium

**Affiliations:** 1 Laboratoire d'Ecologie Alpine, CNRS-UMR 5553, Université Joseph Fourier, Grenoble, France; 2 Natural Resources Faculty, University of Guilan, Guilan, Iran; 3 Environmental Sciences Department, Gorgan University of Agriculture and Natural Resources, Gorgan, Iran; 4 Animal Science Department, Faculty of Agriculture, University of Tabriz, Tabriz, Iran; 5 Animal Production Department, Faculty of Agriculture, Minia University, Minia, Egypt; 6 Department of Genetics, Faculty of Veterinary Medicine, Ankara University, Ankara, Turkey; University of Utah, United States of America

## Abstract

**Background:**

From the beginning of domestication, the transportation of domestic animals resulted in genetic and demographic processes that explain their present distribution and genetic structure. Thus studying the present genetic diversity helps to better understand the history of domestic species.

**Methodology/Principal Findings:**

The genetic diversity of domestic goats has been characterized with 2430 individuals from all over the old world, including 946 new individuals from regions poorly studied until now (mainly the Fertile Crescent). These individuals represented 1540 haplotypes for the HVI segment of the mitochondrial DNA (mtDNA) control region. This large-scale study allowed the establishment of a clear nomenclature of the goat maternal haplogroups. Only five of the six previously defined groups of haplotypes were divergent enough to be considered as different haplogroups. Moreover a new mitochondrial group has been localized around the Fertile Crescent. All groups showed very high haplotype diversity. Most of this diversity was distributed among groups and within geographic regions. The weak geographic structure may result from the worldwide distribution of the dominant A haplogroup (more than 90% of the individuals). The large-scale distribution of other haplogroups (except one), may be related to human migration. The recent fragmentation of local goat populations into discrete breeds is not detectable with mitochondrial markers. The estimation of demographic parameters from mismatch analyses showed that all groups had a recent demographic expansion corresponding roughly to the period when domestication took place. But even with a large data set it remains difficult to give relative dates of expansion for different haplogroups because of large confidence intervals.

**Conclusions/Significance:**

We propose standard criteria for the definition of the different haplogroups based on the result of mismatch analysis and on the use of sequences of reference. Such a method could be also applied for clarifying the nomenclature of mitochondrial haplogroups in other domestic species.

## Introduction

More than 10,000 years ago, the transition of humans from hunting to the manipulation of the behavior of certain animals lead to the process of domestication [1]. This process contributed to the rise of human civilization by enabling people to settle into a sedentary lifestyle. The goat was one of the first domesticated animals [2]–[4]. It was a source of milk, meat, dung for fuel and materials for clothing and building such as hair, bone and skin [1], [5]. Archaeological studies suggested that the domestic goat *Capra hircus* was domesticated from the bezoar *Capra aegagrus* in the Fertile Crescent [e.g. 6]–[8]. This origin was confirmed by genetic studies based on mitochondrial [e.g. 9], [10] and nuclear DNA [11].

From the beginning of the domestication process, the exchange and transportation of domestic animals has been related to human migration and trade. This resulted in genetic (e.g., selection, gene flow) and demographic processes that explain the present worldwide distribution of more than 300 different breeds of *Capra hircus* and their genetic structure [2]. Thus, the present genetic diversity bears the molecular signature of past events, such as rapid demographic expansions. Therefore, the study of this diversity helps to reconstitute the evolutionary history of the goat [12] and could bring new facts that help to understand the history of domestication.

Mitochondrial DNA is commonly used for the study of domesticated species. The control region has been especially used for describing the genetic polymorphism of goats [13], because it is variable and structured enough across the geographical range of the species, and evolves at a constant rate [12]. Moreover, it allows maternal lineages to be followed and is less sensitive to introgression from wild species than nuclear DNA [13]. However, studies on nuclear genes are needed because they give information on gene flow and selection processes that had a great influence on the evolution of livestock species [12].

Luikart et al. [13] conducted the first study of the overall genetic structure of domestic goats at the worldwide scale. They analyzed 406 individuals representing 88 breeds from the old world. They found three mitochondrial haplogroups (A, B and C) that diverged more than 200,000 years ago and have undergone demographic expansion at different times. This would suggest multiple maternal origins of domestic goats or introgression of other haplotypes after the first domestication event. Moreover, they showed that most of genetic diversity occurred within breeds, and interpreted the very weak geographic structure as the result of the extensive transportation of goats among continents.

The initial global survey by Luikart et al. [13] has been followed by regional studies describing more precisely the genetic diversity of goat breeds. However, these studies were always realized in restricted geographic regions corresponding to different countries such as Pakistan [14], India [15], China [16], South Korea [17], Sicily [18], Spain [19], [20] and Portugal [21]. The existence of three new haplogroups has been suggested [14], [15], [18]. However, this has sometimes been based only on a few individuals, and without comparing the new divergent haplotypes to a sample representative of the worldwide haplotype diversity. In general, the identification of a new haplogroup might be controversial in the absence of standardized criteria. All previous studies describing the mitochondrial polymorphism of domestic animals use the term of “maternal lineage” for characterizing a group of closely related haplotypes. However, this term is ambiguous as it usually corresponds to many haplotypes, and thus to many maternal lineages *sensu stricto*. As a consequence, we propose to use “mitochondrial haplogroup” instead of “maternal lineage”, a term that is already in common use in genetic studies.

In this context, the goals of the present study are (i) to characterize the domestic goat mtDNA diversity based on a worldwide sampling and make a global synthesis including previous studies, (ii) to establish the relationships between mitochondrial haplogroups and to propose a clear nomenclature, and (iii) to give standard criteria for the definition of mitochondrial haplogroups. For this purpose we used data from previous studies (1484 sequences retrieved from GeneBank), and we analyzed 946 new samples from all over the old world. New samples were especially taken from localities that have not been adequately sampled before, and that may have played an important role in the history of goat domestication (i.e., Middle East and especially the Fertile Crescent).

## Results

### Sequence polymorphism

The HVI fragment of the control region shows a high polymorphism with 336 variable sites over the 558 bp of the alignment. We observed 285 substitutions (226 transitions and 59 transversions) and 110 insertions/deletions (from 1 to 76 bp). The 2430 individuals correspond to 1540 different haplotypes.

### Phylogenetic analysis and genetic structure of domestic goats

**Figure 1:**
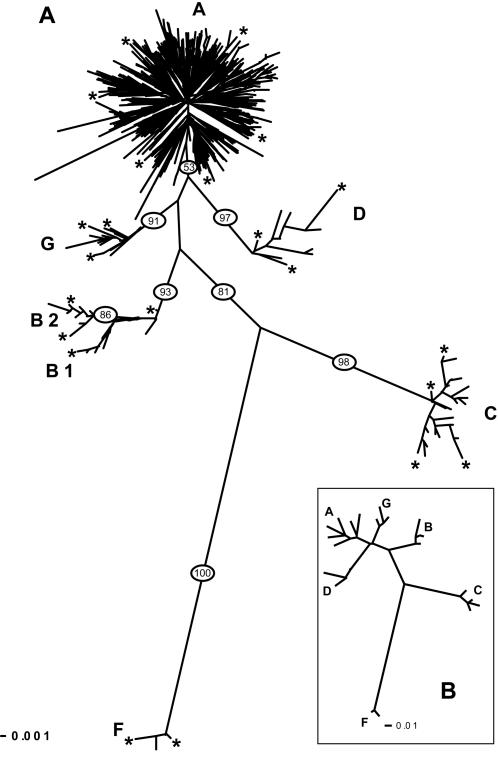
Neighbor-joining trees of domestic goat based on 1540 mtDNA haplotypes (A) and on the 22 reference mtDNA haplotypes (B). Distances were calculated using the Kimura 2-Parameter model with gamma correction (alpha = 0.28). On the (A) tree, the numbers on the branches represent bootstrap values out of 1000 replications, and the stars point out the position of reference individuals for each haplogroup used to construct the (B) tree (see Table 5).

**Figure 2:**
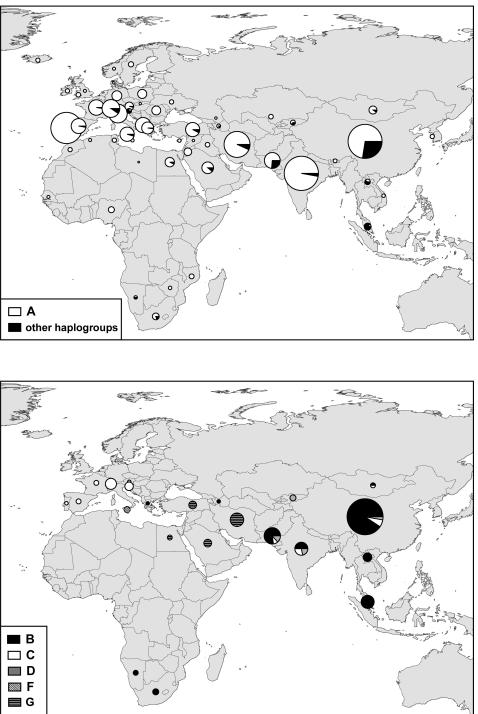
Geographic distribution of domestic goat mtDNA haplogroups. The size of each circle is proportional to the sample size and each specific haplotype is represented by a different colour.

The Neighbor-joining tree of the 2430 domestic goats (Figure 1) shows 6 highly divergent groups corresponding to different mitochondrial haplogroups called A, B, C, D, F (according to previous studies) and G. Each group has high haplotype diversity (Table 1), and has been defined by high bootstrap values (except a bootstrap of 53 % for A ; Figure 1A), and by high mean pairwise distance with all other groups (see below). The A haplogroup is the most represented when considering either the number of individuals or the number of haplotypes and is highly dominant all over the old world (Table 1 and Figure 2). Except for two individuals situated at the base of the B group, this clade is composed of two sub-groups, B1 (35 haplotypes) and B2 (9 haplotypes), as previously defined by Chen et al. [16]. The B group is mostly found in whole Asia, with a few individuals from the Sub-Saharan Africa and one European goat from Greece. The B2 individuals are restricted to China and Mongolia. Goats from the C group are from whole Asia and Europe and the D group is present in the whole Asia and Northern Europe. The three goats from the F group are from Sicily. The G group has not been reported until now, and is present in Middle East and Northern Africa, near the Fertile Crescent.

**Table 1:** Genetic diversity of goat mtDNA haplogroups

haplogroup	# individuals (%)	# haplotypes (%)	haplotype diversity
A	2208 (90.86)	1440 (93.51)	0.9992±0.0001
B	144 (5.92)	46 (2.99)	0.9000±0.0197
B1	107 (4.40)	35 (2.27)	0.8402±0.0333
B2	35 (1.44)	9 (0.58)	0.8151±0.0481
C	35 (1.44)	23 (1.49)	0.9714±0.0136
D	13 (0.54)	10 (0.65)	0.9487±0.0506
F	3 (0.12)	3 (0.19)	1.0000
G	27 (1.11)	18 (1.17)	0.9544±0.0254

The haplotype diversity is very high all over the Eurasia and Africa with a value above 0.97 in 39 of the 54 studied countries (Table 2). More than 77% of the mtDNA variation is distributed within breeds while about 11% is found among breeds within geographic regions and 12 % among geographic regions (Table 3). Nevertheless this low but significant geographic structure is coherent with the fact that all breeds are composed of individuals from the A group, with eventually a lower percentage of individuals from other haplogroups (for about 25% of the breeds). This low geographic structure is also confirmed by the distribution of all haplogroups that are present in several regions (except for F). Most of the mtDNA diversity is distributed among groups and within geographic regions, while less than 4% of this variability is found among regions within groups (Table 3).

**Table 2:** Geographic origin and characteristics of the studied domestic goat

region	country	# of breeds*	# of individuals	# of haplotypes	# ind/haplogroup	Haplotype diversity	Accession numbers
**Eastern Asia (EA)**	Bhutan	1	5	5	A:5	1.0000+/−0.1265	AJ317851-55 (Luikart et al. 2001)
	China	13+U	382	154	A:275; B1: 63; B2: 34; C:7; D: 3	0.9827+/−0.0027	AJ317569-70 (Luikart et al. 2001); DQ089106-13; DQ089116; DQ089135; DQ089147; DQ089155-9; DQ089186-8; DQ089191-209; DQ089212-16; DQ089218-19; DQ089221-2; DQ089237-47; DQ089249-54; DQ089256-57; DQ089269-70; DQ089272-80; DQ089282-304; DQ089350 (Chen et al. 2005); AY853278-301 (Zhang et al. 2004); DQ121491-588; DQ121590-618 (Liu et al. 2006); DQ188849-903 (Liu et al. 2005); AY860871-942 (Zhang et al. 2004)
	Laos	1	10	7	A:4; B1: 6	0.9778+/−0.0540	AB044295- 304 (Mannen et al. 2001)
	Malaysia	1	16	6	A:2; B1:14	0.8583+/−0.0626	AJ317553; AJ317831-32; AJ317828-29 (Luikart et al. 2001); EF618221-31
	Mongolia	2+U	21	21	A:19; B2:1; C:1	1.0000+/−0.0147	AJ317534 -38; AJ317545-52; AJ317833-34 (Luikart et al. 2001); EF618234- 39
	South Korea	U	6	4	A:6	1.0000+/−0.0962	DQ217780- 85 (Lee et al. 2005)
	Vietnam	1	4	3	A:4	0.8333+/−0.2224	AJ317566-68 (Luikart et al. 2001); EF618541
**Middle East (ME)**	Iran	3+U	222	161	A:207; G:15	0.9970+/−0.0008	EF617863- EF618084
	Iraq	1	7	6	A:7	1.0000+/−0.0764	AJ317762-68 (Luikart et al. 2001)
	Jordan	2	19	16	A:19	0.9825+/−0.0223	AJ317769-73 (Luikart et al. 2001); EF618191- 204
	Pakistan	18	73	55	A:56; B1:12; C: 2; D: 3	0.9855+/−0.0076	AJ317533; AJ317539;AJ317554-55; AJ317557-59; AJ317563-65; AJ317826; AJ317845-50; AJ317861-63 (Luikart et al. 2001); AB110552-589(Sultana et al. 2003); EF618253- 63
	Saudi Arabia	3	45	39	A:40; G: 5	0.9949+/−0.0058	AJ317752-59 (Luikart et al. 2001); EF618309- 45
	Syria	1	2	2	A:2	1.0000+/−0.5000	AJ317760-61 (Luikart et al. 2001)
	Turkey	5	66	56	A:61; G: 5	0.9953+/−0.0038	AJ317736-751; AJ317842-43 (Luikart et al. 2001); EF618492- 539
**Western Asia (WA)**	Azerbaijan	1	5	5	A:4; B1:1	1.0000+/−0.1265	EF617702-6
	Dagestan	1	2	2	A:2	1.0000+/−0.5000	EF617708-9
	India	5+U	387	207	A:373; B1:7; C:4; D:3	0.9937+/−0.0008	AJ317827; AJ317856-57; AJ317540-41; AJ317830; AJ317542-44; AJ317560-62; AJ317571-72 (Luikart et al. 2001); AY155674-AY156039 (Joshi et al. 2004); EF617856 - 62
	Kazakhstan	1	7	7	A:7	1.0000+/−0.0764	EF618205- 11
	Kyrgyzstan	1	8	7	A:5; D: 3	0.9643+/−0.0772	EF618212- 19
**Northern Africa (NAF)**	Algeria	1	3	3	A:3	1.0000+/−0.2722	AJ317777-79 (Luikart et al. 2001)
	Egypt	3	29	24	A:27; G: 2	0.9901+/−0.0116	AJ317780-83; AJ317795-801 (Luikart et al. 2001); EF617711- 28
	Libya	U	1	1	A:1	1.0000+/−0.0000	EF618220
	Morocco	1	6	5	A:6	0.9333+/−0.1217	AJ317784 -88 (Luikart et al. 2001); EF618233
	Nigeria	3	12	12	A:12	1.0000+/−0.0340	AJ317810-811; AJ317823-25 (Luikart et al. 2001); EF618246- 52
	Senegal	1	3	3	A:3	1.0000+/−0.2722	AJ317816-18 (Luikart et al. 2001)
	Tunisia	1+U	6	5	A:6	1.0000+/−0.0962	AJ317789-794 (Luikart et al. 2001)
**Sub-Saharan Africa (SAF)**	Mozambique	1	8	5	A:8	0.9286+/−0.0844	AJ317804-809 (Luikart et al. 2001); EF618240- 1
	Namibia	2	4	1	A:2; B1: 2	0.8333+/−0.2224	EF618242- 5
	South Africa	3+U	15	11	A:12; B1: 3	0.9429+/−0.0542	AJ317812-15; AJ317819-20; AJ317844; AJ317821-22 (Luikart et al. 2001); EF618351- 56
	Zimbabwe	1	4	2	A:4	0.8333+/−0.2224	AJ317802-803 (Luikart et al. 2001); EF618545- 6
**Northern Europe (NE)**	Austria	2	24	18	A:23; D:1	0.9783+/−0.0187	EF617678- 701
	Denmark	1	2	1	A:2	1.0000+/−0.5000	AJ317650 (Luikart et al. 2001); EF617710
	England	1	3	2	A:3	0.6667+/−0.3143	AJ317592; AJ317841 (Luikart et al. 2001); EF617729
	France	7	79	61	A:77; C: 2	0.9932+/−0.0039	AJ317575-83; AJ317713-19; AJ317723-25; AJ317629-30 (Luikart et al. 2001); EF617730- 87
	Germany	5	32	25	A:32	0.9919+/−0.0099	AJ317586; AJ317627-28; AJ317649 (Luikart et al. 2001); EF617788- 815
	Iceland	6	6	1	A:6	0.0000+/−0.0000	AJ317587 (Luikart et al. 2001); EF617851- 55
	Ireland	1	6	4	A:6	0.8667+/−0.1291	AJ317588-91 (Luikart et al. 2001); EF618085- 6
	Norway	1	3	3	A:3	1.0000+/−0.2722	AJ317593-95 (Luikart et al. 2001)
	Poland	4	27	22	A:27	0.9943+/−0.0119	AJ317584-85; AJ317651-52 (Luikart et al. 2001); EF618264- 86
	Slovakia	1	2	2	A:2	1.0000+/−0.5000	AJ317653-54 (Luikart et al. 2001)
	Slovenia	1	8	3	A:2; C: 6	0.7143+/−0.1227	AJ317731; AJ317835; AJ317837 (Luikart et al. 2001); EF618346- 50
	Sweden	1	9	7	A:9	0.9722+/−0.0640	AJ317637 (Luikart et al. 2001); EF618415- 22
	Switzerland	11	104	74	A:94; C:10	0.9925+/−0.0026	AJ317573-74; AJ317596-99; AJ317605; AJ317619-24; AJ317626; AJ317631-36; AJ317836; AJ317838- 40; AJ317638-48 (Luikart et al. 2001); EF618423- 91
	Ukraine	1	6	4	A:6	0.9333+/−0.1217	AJ317600-604 (Luikart et al. 2001); EF618540
	Wales	7	7	4	A:7	0.8095+/−0.1298	AJ317655-58 (Luikart et al. 2001); EF618542 - 44
**Southern Europe (SE)**	Albania	6	77	65	A:77	0.9969+/−0.0028	EF617601- 77
	Cyprus	1	4	3	A:4	0.8333+/−0.2224	AJ317774-76 (Luikart et al. 2001); EF617707
	Greece	2+U	47	39	A:46; B1:1	0.9935+/−0.0061	AJ317686-97 (Luikart et al. 2001); EF617816- 50
	Italy	11	115	95	A:115	0.9969+/−0.0018	AJ317674-78; AJ317680-85 (Luikart et al. 2001); EF618087- 190
	Malta	2	4	4	A:4	1.0000+/−0.1768	AJ317659-AJ317702-AJ317708 (Luikart et al. 2001); EF618232
	Portugal	8	321	164	A:320; C:1	0.9941+/−0.0009	AJ317660-69; AJ317698-701; AJ317720-22; AJ317726-30; AJ317732-35 (Luikart et al. 2001); AY961629-697; AY961700- 916 (Pereira et al_2005); EF618287- 95
	Romania	6	26	23	A:26	0.9908+/−0.0133	AJ317606-618 (Luikart et al. 2001); EF618296- 308
	Sicily	1	67	22	A:64; F: 3	0.9701+/−0.0073	DQ241305-71 (Sardina et al. 2006)
	Spain	9+U	73	59	A:71;C:2	0.9962+/−0.0032	AJ317625; AJ317670-73; AJ317679; AJ317703-4;AJ317705-7; AJ317709-12 (Luikart et al. 2001);EF618357- 414

**Note**-U*: Presence of individuals from undefined breed(s)

**Table 3:** Partition of the genetic variance among haplogroups, breeds and continental regions revealed by hierarchical AMOVAs

Source of variation	AMOVA haplogroups/regions			AMOVA regions/breeds		
	Among haplogroups	Among regions within haplogroups	Within regions	Among regions	Among breeds within regions	Within breeds
d.f.	5	20	2404	6	166	1429
% of variation	74.62	3.56	21.82	12.06	10.79	77.14
*P* value	<0.0001	<0.0001		<0.0001	<0.0001	

### Demography of mitochondrial haplogroups

**Figure 3:**
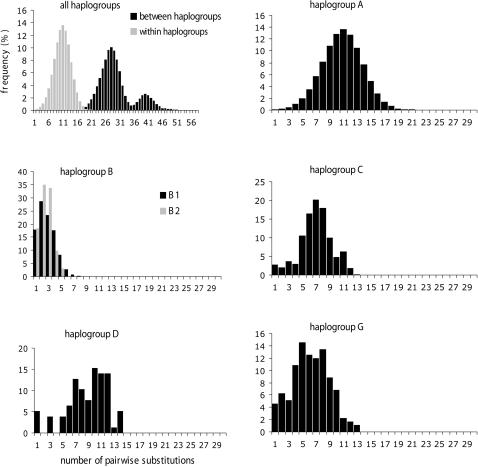
Mismatch distributions for mtDNA haplogroups of domestic goats. For the overall dataset, the distribution of pairwise differences were realized separately for comparisons between and within haplogroups.

Because of the low number of goats in the F group, demographic parameters were not estimated for this group. The overall mismatch distribution shows a multi-modal distribution (Figure 3). The first peak with a maximum of 10 pairwise differences corresponds to the differences between haplotypes from the same group. Two other peaks with maxima at 27 and 39 pairwise differences correspond to differences between haplotypes from different groups. The distributions of within-groups and between-groups pairwise differences have a very thin overlap around 20 mismatches. The mismatch distribution analysis reveals a unimodal bell-shaped distribution of pairwise sequence differences for all haplogroups (Figure 3), except for B that is bimodal (data not shown). B1 and B2 are unimodal, and individuals from these sub- groups generally differ by 8 or 9 mismatches (always less than 14 mismatches). This unimodal pattern that is less clear for the D group, perhaps because of the low sample size (n = 13), would be coherent with recent demographic expansions. The time of expansion would differ according to the group, as suggested by the different means of pairwise distribution (Figure 3) and the estimations made under a model of pure demographic expansion [22] (Table 4). However, the validity of the expansion model used for estimating the expansion time is only accepted for the A, C groups (SSD *P*-Values <0.00001 and ≤0.05 respectively see Table 4). All groups have high growth rates indicating high demographic expansion (Table 4). The estimates differ according to the groups, but the overlapping of confidence intervals, as well as the different sample sizes, preclude further interpretation.

**Table 4:** Estimation of demographic parameters from genetic data

haplogroups	τ (0.95 CI)	Validity of the expansion model SSD (*P*-value)	Rough estimation of Expansion time	Growth rate (0.95 CI)
A	10.07 (9.74–10.42)	0.00071 (***P*** **<0.0001**)	∼9000–9700	308 (199–344)
B1	1.855 (0.73–3.19)	0.0008 (*P* = 0.70)	-	333 (201–412)
B2	1.584 (1.10–2.65)	0.0095 (*P* = 0.20)	-	108 (14–324)
C	6.37 (4.99–7.84)	0.00795 (***P*** ** = 0.05**)	∼4600–7300	185 (158–291)
D	9.10 (5.50–13.01)	0.0141 (*P* = 0.20)	-	334 (173–509)
G	5.79 (2.85–11.22)	0.0021 (*P* = 1.00)	-	209 (144–293)

Note. - See material and methods for the methods used for estimating the demographic parameters. CI: Confidence Interval. SSD: sum of square deviations between the observed and the expected mismatch distributions.

## Discussion

### High mtDNA diversity in domestic goat

The very high mt DNA diversity may partly result from a high mutation rate of the control region. Higher pedigree divergence rates than phylogenetic divergence rates have been shown for the control region in human [23] and other animals (e.g.,[24], [25]). This could explain that we observe a higher diversity than the one expected with the phylogenetic mutation rate estimated for Bovidae (i.e., 30.1 % of divergence per Myr on the total control region sequence based on the *Bos*/*Bison* divergence [26]). Such high variability could also result from the selection of polymorphism but, to our knowledge, this has never been shown for the control region. Another explanation would be the capture of a large part of the diversity of the wild ancestor (i.e., the bezoar) during the domestication, with a large maternal effective population size. Testing this last hypothesis requires comparing the diversity of goats to that of the bezoar [27].

### Characteristics and nomenclature of mitochondrial haplogroups

Five reliable mitochondrial haplogroups have previously been described in domestic goats [13]–[15], [18]. However, most of the previous studies were based on local samples and thus only considered a part of the whole haplotype variability. Therefore, it may be difficult to assess the pertinence of defining a new group on the base of few haplotypes. It would also be difficult to make the correspondence between several studies analyzing samples from different geographic origins. Our study can lead to a clear nomenclature of goat mitochondrial haplogroups, because we analyzed 2430 goats representing 1540 different haplotypes from all over Africa, Asia and Europe (946 new sequences mainly from the region of domestication and 1484 sequences from previous studies). We revealed the existence of 6 highly divergent groups. Five of them (A, B, C, D and F) have already been described, and one (G) is a new group. The two sequences that have been previously used to define the E group [15] now fall within the A haplogroup. This is partly due to the finding of new haplotypes, which are intermediate between those from A and E used by Joshi et al. [15]. Therefore, the E group cannot be considered as a mitochondrial haplogroup anymore. The B clade is composed of two groups (B1 and B2) that have previously been described as “sub-lineages” by Chen et al. [16]. We agree that the B1 and B2 are part of the same haplogroup because the genetic divergence between them (pairwise differences always lower than 14 mismatches) is lower than the divergence between all pairs of haplogroups (more than 20 mismatches). They must be considered as two subgroups because even with a low divergence they are supported by robust bootstrap values.

### Standard criteria for defining goat mitochondrial haplogroups

More generally, previous considerations point out the problem of defining groups and sub-groups. A new haplogroup is defined when it highly diverges from all other haplotypes. However, the haplogroups may change over time, as more and more haplotypes will be available. We faced this situation for the E haplogroup that is no valid any more. There is therefore a need for standard and easy-to-use criteria in order to assign new goat haplotypes to existing haplogroups or to define new haplogroups. A haplotype can be related to an existing group if it presents a moderate genetic divergence from this group. The difficulty may be to define what is a “moderate” divergence. It can be deduced from the distributions of pairwise sequence differences within and between haplogroups. For goats, almost all haplotypes from the same group differ by less than 20 mismatches (whatever the group) while haplotypes from different groups usually present more than 20 mismatches (Figure 3). This threshold value would give a quick and easy indication for almost all studied haplotypes. However, it may be inadequate for some haplotypes (about 1% in our study) because the two mismatch distributions overlap.

Given the increasing number of sequences available, analyzing new haplotypes together with all previously published sequences will be time consuming and will require huge computational resources. Moreover several programs cannot be used because the algorithm complexity does not allow managing such datasets. Especially when a few haplotypes from restricted localities are studied, their assignation to haplogroups should be quick and easy. For a first approach, an accurate solution would be to place the new different haplotypes in a phylogenetic tree containing sequences of reference representative of the diversity of *C. hircus* mitochondrial DNA. For this purpose we have selected 22 haplotypes representing the variability of the 6 present goat mitochondrial haplogroups (Table 5 and Figure 1B).

**Table 5:** The 22 reference individuals of the 6 domestic goat haplogroups

haplogroup	Geographic origin (Country)	Accession Number	Reference
A	India	AY155721	Joshi et al. 2004
A	Italy	EF618134	This Study
A	France	EF617779	This Study
A	Jordan	EF618200	This Study
A	Iran	EF617945	This Study
A	Iran	EF617965	This Study
B1	Laos	AB044303	Mannen et al. 2001
B1	Azerbaijan	EF617706	This Study
B2	Mongolia	AJ317833	Luikart et al. 2001
B2	China	DQ121578	Liu et al. 2006
C	India	AY155708	Joshi et al. 2004
C	Switzerland	AJ317838	Luikart et al. 2001
C	Spain	EF618413	This Study
C	China	DQ188892	Liu et al. 2005
D	India	AY155952	Joshi et al. 2004
D	Austria	EF617701	This Study
D	China	DQ188893	Liu et al. 2005
F	Sicily	DQ241349	Sardina et al. 2006
F	Sicily	DQ241351	Sardina et al. 2006
G	Iran	EF618084	This Study
G	Turkey	EF618535	This Study
G	Egypt	EF617727	This Study

Four of the 1540 haplotypes present a tandemly repeated sequence of 76 bp. Three individuals are from the A group (from Iran, Morocco and India) and one from the B1 sub-group (Malaysia). Such tandem repeats are common in vertebrate species [28] and have already been found in the Bovidae family [29]. They are attributed to slippage-mispairing events that are more likely to appear in regions where the polymerase activity is interrupted [28]. This phenomenon corresponding to a single duplication event is found in a few individuals from different haplogroups, and has occurred more than once in the history of goats.

### Genetic structure of domestic goats

Our results show that most of the genetic variation is found among goat haplogroups, with a weak phylogeographic structure. The strongly dominant A group (91 % of the goats) is distributed worldwide, and even if the other groups have more restricted distributions they still occupy large geographic areas (Figure 2). The F group is the exception, with three haplotypes restricted to a single locality (Sicily) that could have been brought along from recently captured wild goats. However, the sampling effort may still be insufficient to see the whole distribution of haplogroups other than A, because of their low frequency. The differences among geographic regions at the worldwide scale are low (about 12%) but significant. This is concordant with previous results showing a very weak phylogeographic structure of goats [13] and sheep [30], [31] compared to cattle [32], [33]. The genetic differences among continental regions could partly result from the differential geographic distribution of mitochondrial haplogroups. However, there is still a low but significant genetic variation (3.5%) among region within groups, indicating regional differentiations of haplotypes. At the regional scale, the lack of geographic structure has also been reported in several places [16], [19], [21] while a structure has been found in India [15]. The weak phylogeographic structure found today in goats has been explained by a high mobility of this species in relation to human migration and commercial trade [12], [13], [34]. This mobility would have been higher than those of cattle due to their versatility in feeding habits and ability to live under extreme conditions [1]. However, the mixing of goat haplogroups could have existed before the worldwide translocation of goats. The presence of goats in Cyprus 10,000 years ago [35] suggests that goats could have been translocated within the domestication area since the first domestication events. Moreover, we cannot exclude that the mt-haplogroups were already mixed in the wild ancestor before domestication. When considering the local scale, the genetic pattern of domestic goats also seems related to human history. For instance, the geographic structure found in Indian goats would have a common historical basis in the sequential migrations of human populations with different cultural and linguistic characteristics [15].

However, the information given by mitochondrial markers is limited because it does not detect male-mediated gene flow and does not predict the nuclear genomic diversity [12]. In particular, the breeds cannot be distinguished on the base of mtDNA [16], [19], [20] while nuclear markers show a genetic structure [36]–[38]. Our study confirmed that more than 77 % of the mtDNA variation is found within breeds and that nearly 25% of the breeds are composed of at least 2 haplogroups. This is in accordance with the recent fragmentation of local goat populations into discrete breeds about 200 years ago, under strong selection pressures on a few phenotypic traits [39]. This structure can be seen on nuclear markers linked to selected parts of the genome, but not on mitochondrial markers. Then, looking at the evolutionary history of breeds using mtDNA markers could lead to misinterpretation. For example, a breed composed of two mitochondrial haplogroups would have a bimodal mismatch distribution due to within- and between-breeds pairwise differences, and should not be interpreted in term of demographic history of the breed. Thus, fully understanding the evolutionary history of domestic goats would also require the use of nuclear markers.

### Demography of mitochondrial haplogroups

The present structure of the genetic diversity retains the signature of past demographic events and helps reconstitute the evolutionary history [40]. The estimation of demographic parameters remains difficult because of the difficulties of verifying the hypothesis of the models used, of estimating accurate initial parameters (e.g., absolute date of domestication) and sometimes because of low sample sizes. However, rough estimations from the present work and previous studies [13], [15], [16] are concordant and agree on the same scenario. All haplogroups had a recent demographic expansion corresponding roughly to the period when domestication took place about 10,000 years ago. It is difficult to give relative dates of expansion because of large confidence intervals, especially for D and G groups, but our results confirm that the expansions of B and C groups were more recent than that of A [13]. Also, our results show that all groups had high growth rates, with a tendency for slower growth in B2 sub-group and C and G. A faster growth of A relative to C is in accordance with archaeozoological data: the genotyping of fossil goats showed that about 7000 years ago A and C were equally represented in Southern France [34] while A is strongly dominant in Southern Europe now.

### Limits of genetic data from domestic goats for reconstituting the history of domestication

Divergence time between haplogroups has been estimated on adequate molecular markers (mainly cytochrome *b*) between 103,000 and 597,800 years [13]–[16]. All these values are far greater than the domestication time, showing that most of goat genetic diversity existed before domestication, and that several haplogroups were domesticated in one or several events. However, the genetic data available for domestic goats does not permit furthering our understanding of the domestication process and identifying potential domestication centre(s). A higher genetic diversity would have been expected near the Fertile Crescent where the goat domestication took place according to archaeological data, and where extensive sampling has been done. But the haplotype diversity is similar all over the world (more than 80% of the countries with a haplotype diversity greater than 0.9), because of the high migration rates in domestic goats due to human migration and commercial trade.

Moreover, the presence of a possible ancestral haplotype in a particular area does not prove that this is a domestication centre, since many events could have occurred to mask the real history (e.g., coalescence or founder effects). For instance the domestication of a B sub-group in China supported by genetic data [16] is doubtful since the wild ancestor of the domestic goat (i.e. the bezoar *Capra aegagrus*) has credibly never been present in this area [11], [41]. Overall, in order to fully understand the domestication of goats it is necessary to characterize the genetic diversity of wild goat species, and to establish the evolutionary relationships between wild and domesticated haplotypes.

## Materials and Methods

### Sampling and DNA extraction

Samples were collected from 946 individuals from 42 countries of the old world (See Table 2) from which 569 individuals were studied within the Econogene project (www.econogene.eu). Samples consisted of ear tissue preserved in ethanol 95% until extraction, or of blood collection. DNA was extracted from tissue using the Qiagen DNeasy tissue kit following the manufacturer's instructions, and from blood samples using QIAamp DNA blood kit.

To have a good coverage of the goat breeds, the dataset was completed with 1484 sequences containing the *Capra hircus* HVI control region (450 to 1200 bp long) retrieved from GenBank (Table 2).

### DNA amplification and sequencing

The HVI segment of the control region was sequenced for all blood and tissue DNA extracts. Using the primers CAP-F (5′-CGTGTATGCAAGTACATTAC-3′) and CAP-R (5′-CTGATTAGTCATTAGTCCATC-3′), we amplified a fragment of 598 bp (without primers) that corresponds to the positions 15,653 to 16,250 on the complete goat mitochondrial sequence of reference ([42]; accession number AF533441). PCR amplifications were conducted in a 25 µl volume with 2 mM MgCl_2_, 200 µM of each dNTP, 1 µM of each primer and 1 unit of AmpliTaq Gold Polymerase (Applied Biosystems). After a 10 min period at 95°C for polymerase activation, 35 cycles were run with the following steps: 95°C: 30 s, 55°C: 30 s, 72°C: 1 min. PCR products were purified using the Qiaquick PCR purification kit (Qiagen). 35 ng of purified DNA from this PCR product was used for sequencing with the CAP-F or CAP-R primer. Sequence reactions were performed for both DNA strands by using the ABI PRISM Dye Terminator Cycle Sequencing Reaction Kit (Applied Biosystems) in a 20 µl volume with 2 µM of each primer. 25 cycles were run with the following steps 96°C: 30 s, 55°C: 30 s, 60°C: 4 min. Excess dye terminators were removed by spin-column purification and the products were electrophorezed on an ΑΒΙ 3700 PRISM DNA sequencer (Applied Biosystems) using the POP 7 polymer.

Sequences were edited for correction with the SeqScape v2.5 software (Applied Biosystems). All sequences were deposited in GenBank (Accession Numbers EF617601- EF618546, Table 2 and Table S1).

Sequences from GenBank and from our dataset were aligned with Mega v3.1 [43], and then adjusted by eye. For further analyses, we only kept the region used by Luikart et al. [13] because this is the part of the sequence available for most of the GenBank records, and also the most informative one. This region is 481 bp long and corresponds to the positions 15,707 to 16,187 on the *Capra hircus* reference sequence (mtDNA complete sequence of *C. hircus*, Accession number AF533441 [42]). According to the insertion/deletion events, the analyzed sequences ranged from 481 to 558 bp. For Indian goats a shorter fragment of 453 bp has been sequenced [15] and the 28 missing nucleotides were treated as missing data. The alignment of the 2430 sequences used in this study is provided as supplementary information (Table S1).

### Data analysis

The substitution model used for the HVI region was the Kimura 2-parameters model, as previously used on several subsets of the present dataset (e.g., [13], [15]). The heterogeneity in substitution rates among nucleotide sites was modelled by a gamma distribution. The alpha parameter was estimated as the mean of 10 estimations by a maximum-likelihood method under the Kimura 2-parameters model using PAML v 2.0.2 [44]. Each estimation was based on the analysis of 1000 individuals randomly chosen in the dataset of 2430 individuals. The estimated value (alpha = 0.28) was similar to that estimated for the same region on a smaller sample of domestic and wild goats by Luikart et al. [13]. These settings were used for further phylogenetic reconstruction and analysis of genetic diversity. We used 1484 published sequences for checking the validity of the halpogroups previously defined (see Table 2 and Table S1 for references and GenBank accession numbers).

Given the very high number of sequences analyzed, the phylogenetic tree was constructed using the Neighbor-joining method using PAUP* v 4.0 [45], with 1000 bootstraps for measuring branch robustness. The ARLEQUIN v 3.0 software [46] was used for estimating haplotype and nucleotide diversity, for analyzing mismatch distribution within mitochondrial haplogroups, and for estimating the parameters of demographic expansion. Four individuals that showed a 76 bp insertion were discarded for mismatch analyses and the analyses were thus performed on 481 bp long sequences. The expansion time was estimated under a model of pure demographic expansion [22] with parameters set to default values in ARLEQUIN 3.0. The parameter of demographic expansion τ was estimated according to the method of Schneider and Excoffier [47]. The validity of the expansion model was tested using the sum of square deviations (SSD) between the observed and expected mismatches [47]. Growth rates of mitochondrial haplogroups were estimated with Lamarc v2.2 [48] using a bayesian framework allowing migrations across haplogroups (with a maximum of 10000 migration events, default priors used for migration rates estimation). The estimation of growth rates was done with linear prior (upper bound of 1000 and lower bound of −500), 10 initial chains (500 samples, sampling interval of 20 and burn-in period of 1000) and 2 final chains (10000 samples, sampling interval of 20 and burn-in period of 1000).

In order to test the geographic structure of the mtDNA haplotype diversity, the goat distribution has been partitioned in 7 geographic regions (Northern Europe, Southern Europe, Northern Africa, Sub-Saharan Africa, Middle East, Western Asia and Eastern Asia, see Table 2). Two hierarchical AMOVA were performed using ARLEQUIN v3.0 to test the partition of the genetic variance among haplogroups and among continents within haplogroups, as well as among continents and among breeds within continents. This second AMOVA was performed on the 1602 goats for which the breeds were known.

## Supporting Information

Table S1 Alignements of the 2430 control region sequences of domestic goat. The code for geographic regions are defined in Table 2. Missing data are coded as ‘?’.
(1.24 MB XLS)
